# The chimeric multi-domain proteins mediating specific DNA transfer for hepatocellular carcinoma treatment

**DOI:** 10.1186/s12935-016-0351-0

**Published:** 2016-10-13

**Authors:** Encheng Yang, Xiao Li, Ningyi Jin

**Affiliations:** 1 Department of Gastroenterology and Hepatology, The Second Affiliated Hospital of Harbin Medical University, Harbin, 150086 China; 2 Institute of Military Veterinary, Academy of Military Medical Sciences of PLA, #666 Liuying West Road, Jingyue District, Changchun, 130122 Jilin province China

**Keywords:** Hepatocellular carcinoma, Tat-apoptin, Antitumor effect, HepG-2, Mouse model

## Abstract

**Aim:**

This study was aimed to evaluate the therapeutic efficiency of a non-virus based specific chimeric multi-domain DNA transferred with apoptin in human hepatocellular carcinoma (HCC) HepG-2 cells in vitro and in mice H22 cells in vivo.

**Methods:**

We firstly constructed the multi-domain recombinant chimeric proteins based on recombinant proteins [G (yeast GAL4), NG (none GAL4), TG (GAL4 + Tat protein) and TNG (Tat protein)] and pUAS-Apoptin plasmid, and transfected them into human HepG-2 cells. The antitumor effect of this multi-domain recombinant chimeric proteins to HCC cells were detected by MTT assay, AO/EB staining, DAPI staining and Annexin V assay. In order to find the pathway of cell apoptosis, the Caspase (1, 3, 6 and 8) activity was detected. We then constructed the H22 liver cancer mice model and analyzed the anti-tumor rate and mice survival rate after treated with G/pUAS-Apoptin NG/pUAS-Apoptin TG/pUAS-Apoptin, and TNG/pUAS-Apoptin.

**Results:**

MTT results showed that the Tat protein (TG and TNG) significantly induced cell death in a time dependent manner. AO/EB, DAPI, Annexin V and Caspases assay results indicated that the Caspase 1, 3, 6 and 8 were highly expressed in TG/pUAS-Apoptin, and TNG/pUAS-Apoptin treated mouse groups. The antitumor rate and survival rate in TG/pUAS-Apoptin, and TNG/pUAS-Apoptin treated mouse groups were higher than in the other groups.

**Conclusion:**

The Tat-apoptin is a potential anti-tumor agent for HCC treatment with remarkable anti-tumor efficacy and high safety based on non-virus gene transfer system. The anti-tumor function may be associated with high expression of Caspase 1, 3, 6 and 8.

**Electronic supplementary material:**

The online version of this article (doi:10.1186/s12935-016-0351-0) contains supplementary material, which is available to authorized users.

## Background

Hepatocellular carcinoma (HCC) is a slowly developing malignant tumor that evolves from premalignant lesions in a chronically damaged liver [1–3]. HCC is a late complication in patients with chronic liver disease, and therefore, is often associated with cirrhosis and mortality [4, 5]. One of the major reasons for the high mortality in HCC patients is the limited treatment options and poor prognosis [6]. Traditional treatments of HCC, such as operation, chemotherapy and radiotherapy, bear some side effects and limit the dose that can be administered [7, 8]. Therefore, developing effective cancer therapeutic strategies is of particular importance for patients with HCC.

In the past decades, a line of evidences indicate the promising potential of gene therapy for HCC treatment [9, 10]. As a process of gene transfer, gene therapy is largely relied on the development of gene delivery vector. Nowadays, there are several kinds of gene delivery systems that have been explored, such as modified siRNA, viral vectors and non-viral vectors [11]. Viral vector has been widely used for the high efficiencies of transfection and foreign gene expression, but it shows advantages in oncogenic effects, endogenous virus and unexpected immune response. Non-viral vector has gained substantial interest for the flexibility in design and non-infectivity [12, 13].

GAL4 protein is known as a transcriptional activator that can bind to the galactose upstream active sequence (UAS) to stimulate the expression of genes involved in galactose metabolic pathway [14]. A previous study has proposed that GAL4 is a promising vector for non-viral DNA transfection [15]. Viral NLS (nuclear localization sequence) is served as a logical addition to non-viral vector delivery system. The fusion protein GAL4-NLS has been determined to enhance the DNA transfection efficiency to mammalian nuclei [16]. The Tat protein is a transcriptional activator from human immunodeficiency virus type 1 (HIV-1) and shows the ability to translocate through the plasma membrane [17]. In our preliminary study, a novel fusion protein termed TG has been found to be an efficient carrier of DNA plasmid, which contains GAL4 domain as the carrier for DNA plasmid and Tat domain for cell internalization [18]. However, the application of fusion protein as the plasmid carrier in treating HCC cells has not been reported.

Apoptin is a small apoptosis protein that derived from chicken anemia virus. Gene therapy experiments have proved that apoptin expression may cause tumor regression and cell apoptosis without any significant side effect [19–21]. In this study, the multi-domain recombinant chimeric proteins such as G (GAL4), NG (NLS-GAL4), TG (Tat-GAL4 protein) and TNG (Tat-NLS-GAL4 protein), and pUAS-Apoptin plasmid were constructed. We attempted to evaluate the inhibitive effect on HCC by systemic delivery of Apoptin gene via multi-domain recombinant chimeric proteins.

## Methods

### Construction of recombinant plasmid

The 17-mer upstream activating sequence (UAS) was obtained by tandem synthesis and then cloned into the plasmid vector pIRESneo (Invitrogen, USA) at the *Xho*I site to construct the recombinant plasmid pUAS. The recombinant plasmid of chimera pUAS-EGFP was constructed by double digestion of plasmid vector pMT-EGFP and pUAS with restriction endonuclease *Not*I and *Xba*I. Similarly, the recombinant plasmid pUAS-Apoptin was constructed and identified by double digestion of pMT-Apoptin and pUAS with endonuclease *Eco*RI and *Eco*RV. The structure of the plasmid used was showed in the Additional file 1: Figure S1.

### Plasmid chimera and fusion protein transfection into human HepG-2 cells

The human hepatoma HepG-2 cells (Chinese Academy of Sciences, China) were routinely cultured at 37 °C in 5 % CO_2_. The schematic representation of transfection-expression strategy was listed in Additional file 2: Figure S2.

Before pUAS-EGFP transfection, HepG-2 cells (1 × 10^5^ cell/well) were seeded in a 96-well plate and cultured until 80 % confluence. These cells were transfected with 10 μl of recombinant pUAS-EGFP and 32 μl of fusion protein G, NG, TG and TNG (constructed in our previous study [18]) followed by culturing for 48 h at 37 °C under 5 % CO_2_. The cells were then observed by fluorescence microscope (Olympus, Japan). The equal volume of normal saline treated cells and the plasmid/lipidosome complex cells were used as negative control and positive control, respectively.

For pUAS-Apoptin transfection, cells were plated at 5 × 10^3^ cell/well in a 96-well plate, and then transfected with 1 μl of pUAS-Apoptin recombinant and 3.2 μl of G, NG, TG and TNG, respectively. Subsequently, the cells were cultured at 37 °C under 5 % CO_2_. Cells that were cotransfected with liposome and pUAS-EGFP were served as positive controls.

### MTT assay

The 3-(4,5-dimethyl-thiazol-2-yl)-2,5-diphenyltetrazolium chloride (MTT) assay was conducted for evaluating the effects of TG/pUAS-Apoptin and TNG/pUAS-Apoptin on HepG-2 cell proliferation. In brief, after transfection for 12, 24, 36, 48, 60, 72, 84 and 96 h, the transfected cells were treated with 20 μl MTT (5 mg/ml, Sigma, USA) per well for 4 h. After that, the liquid supernatant of culture medium was discarded, and 150 μl DMSO was added per well to dissolve the precipitate. Absorbance was measured at 490 nm (A_490_) with normal saline treated cells as controls. Suppression rate as calculated as follows: ${\text{suppression rate }}\left( \% \right) = ( 1 - {\text{A}}_{ 490}\, {\text{in treatment group}}/{\text{A}}_{ 4 90} \,{\text{in control group}}) \times 100 \;\%$.

### Apoptosis assay

Apoptosis was evaluated by flow cytometry Annexin V Flous staining. In brief, HepG-2 cells were trypsinized and washed with phosphate buffer solution (PBS) after 60 h of transfection. After centrifuged at 3000 rpm/min for 5 min at 4 °C, 1 × 10^5^ cells, were re-suspended in 195 μl of binding buffer, and added with 5 μl of fluorescein isothiocyanate (FITC)-labeled annexin V (Beyotime, China) for 10 min. Subsequently, the cells were centrifuged and re-suspended in 190 binding buffer followed by dyeing with 10 μl of propidium iodide (PI) for 5 min. Flow cytometry was conducted with the normal saline treated cells as controls. The same experiment was repeated for three times.

### AO/EB and DAPI (4′, 6-diamidino-2-phenylin-dole) staining

AO/EB and DAPI staining were conducted for the observation of cellular morphology. With respect to AO/EB staining, transfected cells were firstly washed and re-suspended with PBS, and then stained with 1 μl of acridine orange (AO, Sigma, USA) and 1 μl of ethidium bromide (EB, Sigma, USA) solution. As to DAPI staining, the transfected HepG-2 cells were washed with PBS and re-suspended with alcohol, followed by 0.5 % Triton treatment for cell permeabilization. Then the cells were collected and stained with DAPI (Sigma, USA) for 15 min.

After staining, the morphologic changes of cells were observed under a fluorescence microscope at 3 random fields (≥300 cells/each field). The saline treated cells were used as normal controls.

### Detection of protein concentration and caspase activity

Protein concentrations were detected by 2,2′-biquinoline-4,4-dicarboxylic acid disodium salt (BCA, Beyotime, China) assay. Briefly, the transfected cells were firstly washed with PBS and treated with lysis buffer for 15 min in ice-water. For protein preparation, the cells were centrifuged at 4 °C for 15 min and the supernatant was collected for the following investigation. The working reagent was prepared by BCA Protein Assay Kit according to the manufacturers’ instruction. The standard sample was fully dissolved and diluted to a final concentration of 0.5 mg/ml with PBS. A serial volume of standard samples (0, 1, 2, 4, 8, 12, 16 and 20 μl), and 20 μl of protein sample were respectively added into the enzyme-linked immunosorbent assay (ELISA) plate. The mixture was supplemented with PBS to 20 μl, and reacted with 200 μl of working reagent. Based on the standard curve, absorbance was measured at 562 nm (A_562_) for protein concentrations calculation.

The enzymatic activity of caspase 1, 3, 6 and 8 was detected with the similar method described above, while the reaction was initiated after adding 2 mM of Ac-YVAD-pNA at 37 °C for 120 min and absorbance was observed at 405 nm.

### Construction of H22 HCC mouse model (C57BL/6)

The 6-week old female C57BL/6 mice were purchased from the People’s Liberation Army Academy of Military Medical Sciences, China. The animal experiments were approved by the Institutional Animal Care and Use Committees and the study procedures were performed in accordance with the guidelines of the National Institutes of Health Guide for the Care and Use of Laboratory Animals. Mice were fed with standard rodent chow and raised under room temperature.

The H22 HCC mouse model was constructed as follows: the mice were firstly intraperitoneally inoculated with H22 cells (obtained from laboratory animal center of Jilin University, China). After 7–10 days incubation, ascites was extracted. H22 cells (1 × 10^7^ cell/ml) in ascites were washed for three times, and subcutaneously injected into the right hind leg of the mice (100 μl, 1 × 10^6^ cells). The model was considered to be successfully constructed when the tumor nodules grew in a diameter of 3–5 mm.

### Grouping and treatment

A total of 54 HCC model mice were randomly divided into nine groups (n = 6 per group), including the normal saline, pUAS, pUAS-Apoptin, TG, TNG, TG/pUAS, TG/pUAS-Apoptin, TNG/pUAS and TNG/pUAS-Apoptin treatment group. Additional six normal mice were enrolled in the control group.

Total 100 μg of corresponding plasmid or fusion protein or combined plasmid and fusion protein (at dosage ratio 1:32) was injected to mice for three times with 10-day interval, which was prior dissolved in 100 μl of saline. Mice in the normal saline treatment group were treated with 100 μl of normal saline solution. All the treated mice were sacrificed at 16 days after the final treatment.

### Detection for survival rate and tumor volume

After treatment, the tumor diameter (D) and thickness (T) were measured by vernier caliper every 2 days. The tumor volume (V) = D × T^2^ × 0.52. ${\text{Anti}} - {\text{tumor rate}} = ( {{\text{V}}_{\text{normal saline}}  {-} {\text{V}}_{\text{treatment group}} } )/{\text{V}}_{\text{normal saline}} \times 100 \;\%$. Simultaneously, survival rate was calculated for each group and Kaplan–Meier curve was used for survival analysis.

### Tissue section analysis

The tumor tissues extracted from mice were fixed in 10 % formalin for 24–72 h, and dehydrated with ethyl alcohol and xylene. Then the tissues were embedded in paraffin and cut into 4-μm-thick sections. The slides were stained with hematoxylin-eosin (HE), and observed by using an light microscope at 40× magnification.

### Statistical analysis

All data were presented as mean ± standard deviation (SD). Comparison between groups was performed with the *t* test. Kaplan–Meier curve was conducted for survival analysis of mice models. P < 0.05 was considered as statistically significance.

## Results

### Construction of recombinant plasmids

The recombinant plasmid pUAS, pUAS-EGFP and pUAS-Apoptin were identified by double digestion with corresponding restriction endonucleases. Finally, DNA fragments with length of approximately 100, 750 and 360 bp were obtained after double digestion of pUAS, pMT-EGFP and pMT-Apoptin, respectively, which determined that the recombinant plasmid of pUAS, pMT-EGFP and pMT-Apoptin were constructed successfully.

### Transfection of fusion proteins and recombinant plasmids into HepG-2 cells

**Fig. 1:**
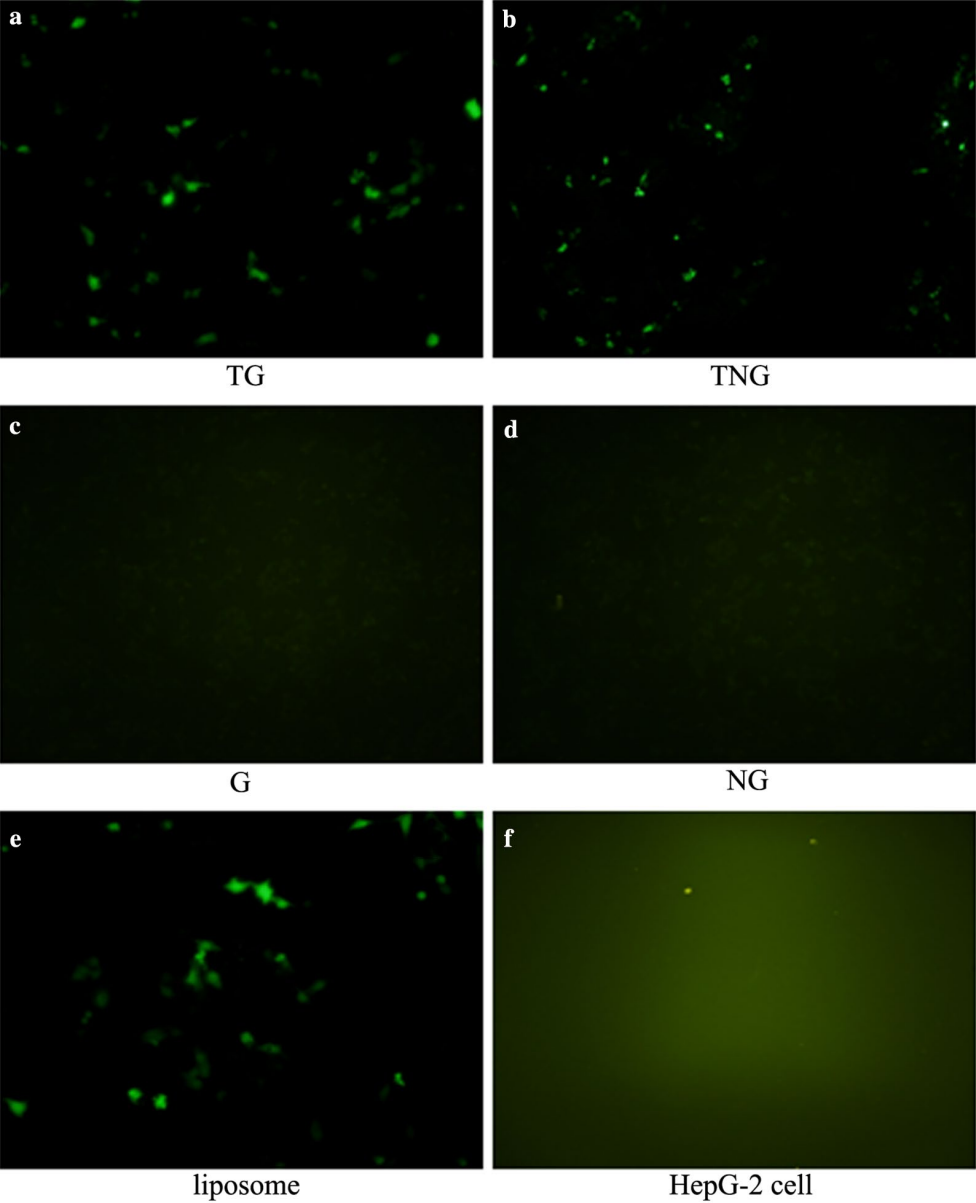
The transfection of the recombinant fused protein in HepG-2 cells observed by fluorescence microscope at ×40 magnification. Green fluorescence was only found in HepG-2 cells transfected with TG/pUAS-EGFP and TNG/pUAS-EGFP for 48 h, as well as with the liposome/pUAS-EGFP. **a** TG/pUAS-EGFP; **b** TNG/pUAS-EGFP; **c** G/pUAS-EGFP; **d** NG/pUAS-EGFP; **e** liposome/pUAS-EGFP; **f** HepG-2 cell

Green fluorescence was only observed in HepG-2 cells transfected with TG/pUAS-EGFP and TNG/pUAS-EGFP for 48 h, as well as with the liposome/pUAS-EGFP. There was no green fluorescence in HepG-2 cells transfected with other fusion proteins and/or recombinant plasmids (Fig. 1).

### Cytotoxicity of pUAS-Apoptin to HepG-2 cells

**Fig. 2:**
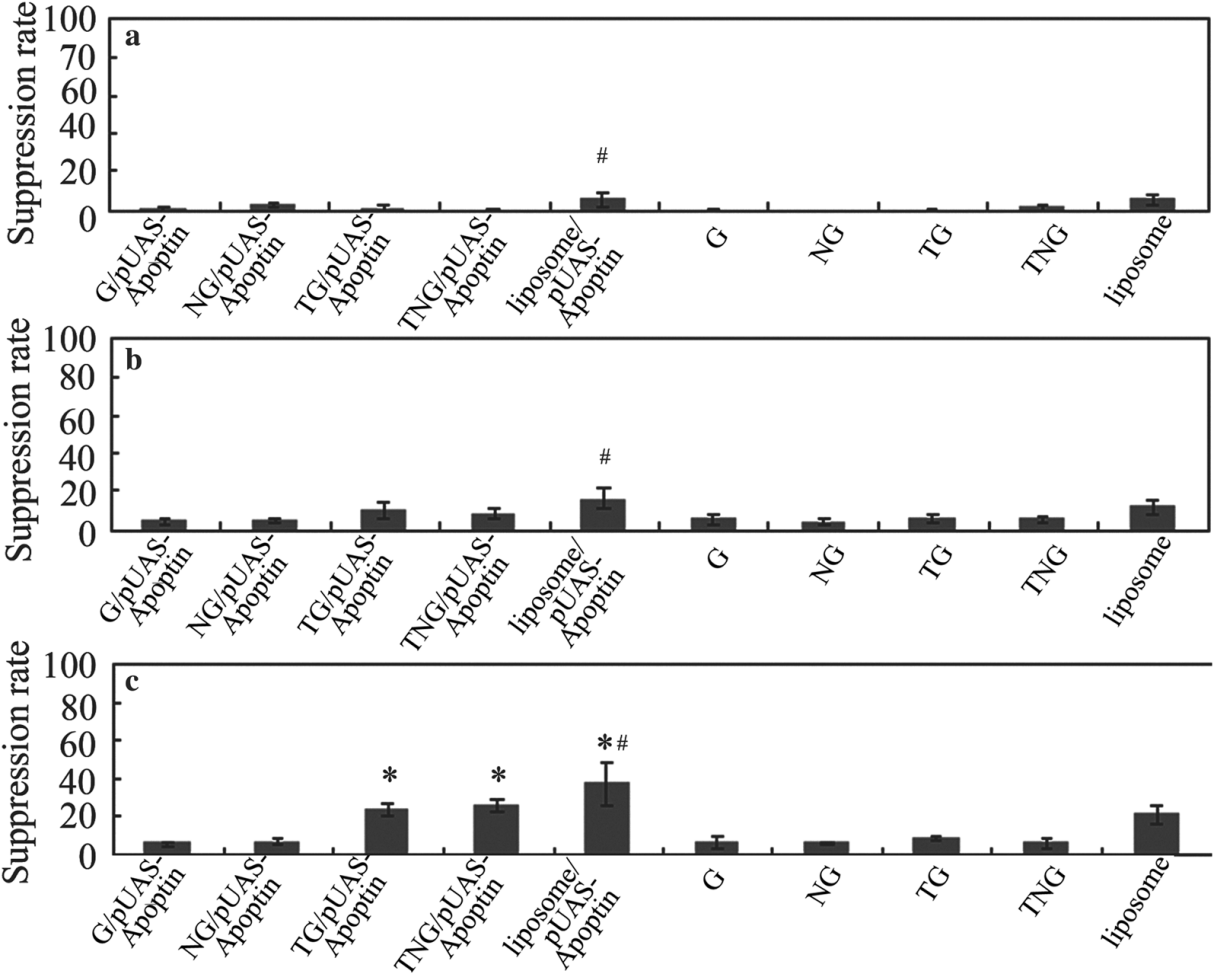
MTT assay for the suppression rate of various treatment to HepG-2 cells after 12 h (**a**), 24 h (**b**) and 36 h (**c**). From the 36 h of incubation, the suppression rates were significantly higher in HepG-2 cells transfected with TG/pUAS-Apoptin, TNG/pUAS-Apoptin and lipidosome/pUAS-Apoptin than that of HepG-2 cells transfected with G/pUAS-Apoptin and NG/pUAS-Apoptin (P < 0.05). *Asterisk* indicates significant difference compared with G/pUAS-Apoptin, NG/pUAS-Apoptin, G, NG, TG and TNG treated cells at *P* = 0.05 level. *Hash* indicates significant difference between the liposome/pUAS-Apoptin with TG/pUAS-Apoptin and TNG/pUAS-Apoptin

The cytotoxicity of pUAS-Apoptin and fusion proteins to HepG-2 cells was detected by MTT staining (Fig. 2). At 12 h (Fig. 2a) and 24 h (Fig. 2b), the suppression rates of HepG-2 cells transfected with TG/pUAS-Apoptin, TNG/pUAS-Apoptin or lipidosome/pUAS-Apoptin showed no significant difference when compared with that of HepG-2 cells transfected with G/pUAS-Apoptin and NG/pUAS-Apoptin (*P* > 0.05). However, at 36 h of incubation (Fig. 2c), the suppression rates were significantly higher in HepG-2 cells transfected with TG/pUAS-Apoptin, TNG/pUAS-Apoptin and lipidosome/pUAS-Apoptin than those transfected with G/pUAS-Apoptin and NG/pUAS-Apoptin (*P* < 0.05), indicating that the TG, TNG and lipidosome significantly mediated pUAS-Apoptin cytotoxicity to HepG-2 cells (the rest graphs for treatment at 48 h (D), 60 h (E), 72 h (F), 84 h (G), and 96 h (H) were showed in the Additional file 3: Figure S3). In addition, HepG-2 cells transfected with lipidosome/pUAS-Apoptin showed higher suppression rate than those transfected with TG/pUAS-Apoptin and TNG/pUAS-Apoptin at 12, 24 and 36 h of incubation (*P* < 0.05). The cytotoxic effect of pUAS-Apoptin induced by TG and TNG reached the peak at 48–60 h of incubation.

### pUAS-Apoptin induced cell apoptosis mediated by TG and TNG

**Fig. 3:**
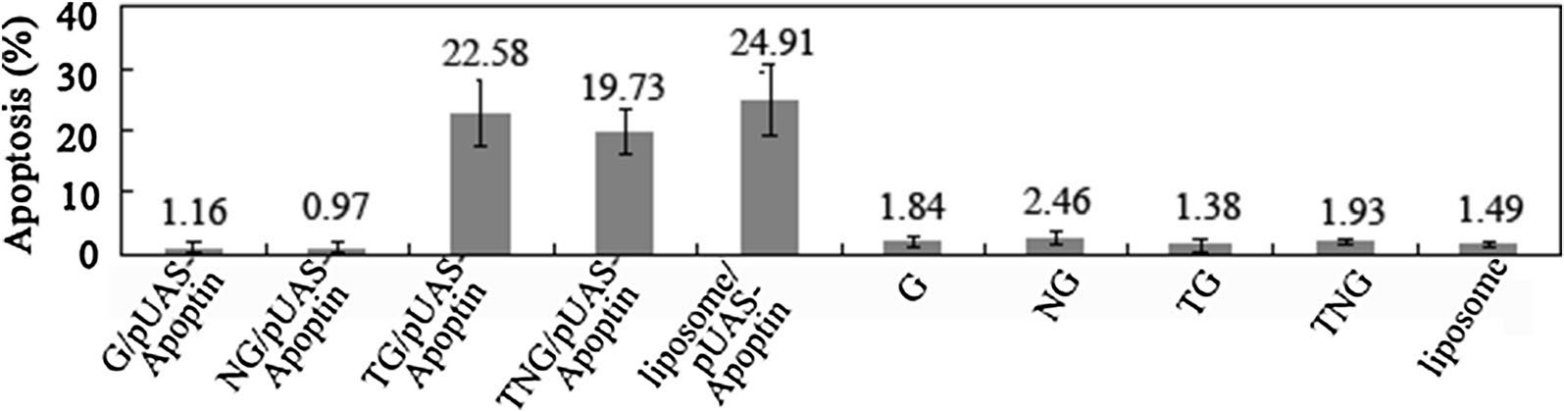
Cell apoptosis detected by Annexin V assay

After treated with FITC-labeled annexin V and PI, HepG-2 cells were tested by flow cytometer for cell apoptosis. The apoptosis rates were 22.58 and 19.73 % for HepG-2 cells transfected with TG/pUAS-Apoptin and TNG/pUAS-Apoptin, respectively, which showed no significant difference with that of HepG-2 cells transfected with lipidosome (24.91 %) (Fig. 3).

### pUAS-Apoptin induced changes in cellular morphology mediated by TG and TNG

**Fig. 4:**
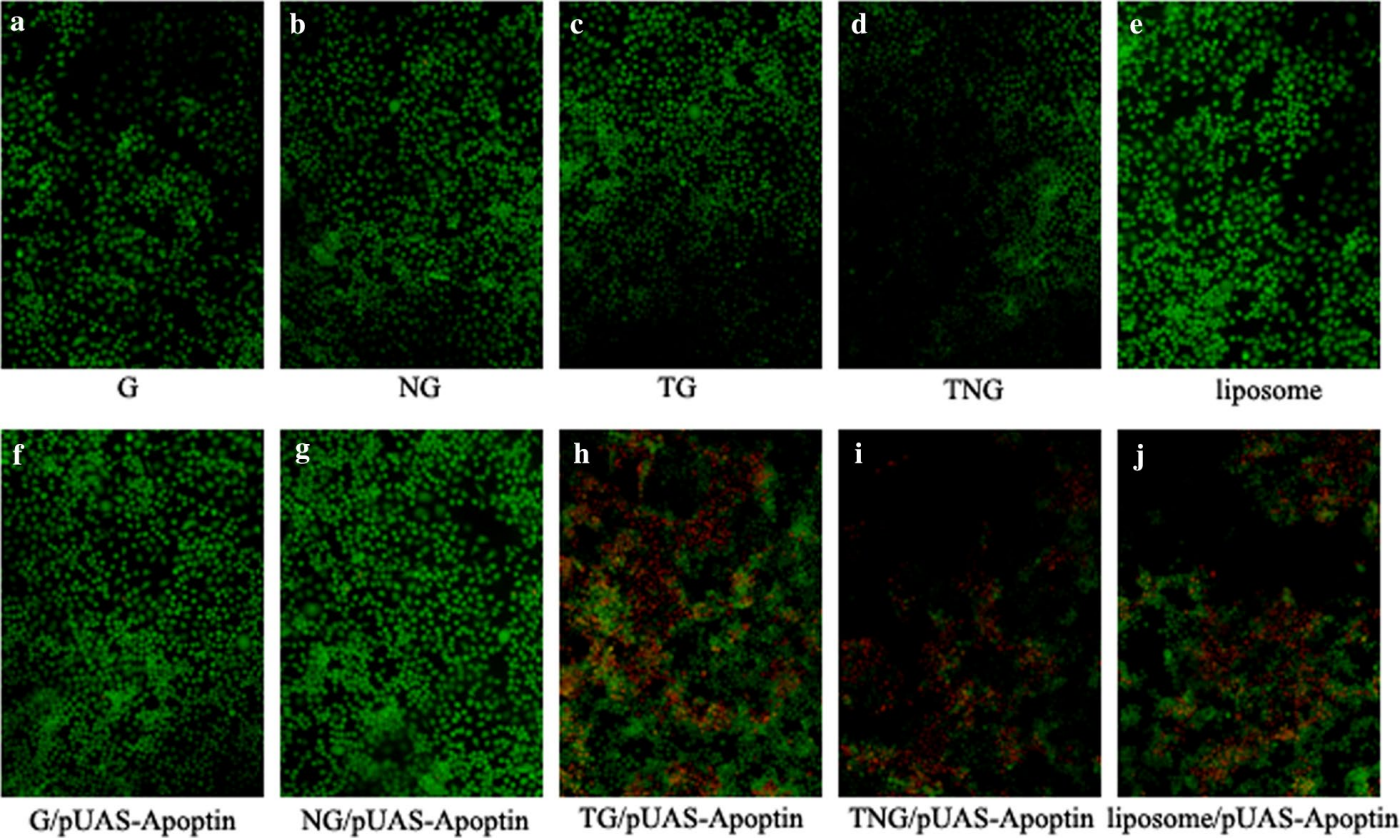
AO/EB staining result of different treated HepG-2 cells (×40). TG/pUAS-Apoptin, TNG/pUAS-Apoptin and lipidosome/pUAS-Apoptin transfected cells exhibited a *red color* in the nucleus, indicating that these cells had lost membrane integrity and dead. Conversely, the G/pUAS-Apoptin and NG/pUAS-Apoptin transfected cells showed a *green color* in the core, indicating that these cells were alive. **a** G; **b** NG; **c** TG; **d** TNG; **e** liposome; **f** G/pUAS-Apoptin; **g** NG/pUAS-Apoptin; **h** TG/pUAS-Apoptin; **i** TNG/pUAS-Apoptin; **j** liposome/pUAS-Apoptin

Changes in cellular morphology was observed by AO/EB staining and DAPI staining. The AO/EB staining showed that TG/pUAS-Apoptin, TNG/pUAS-Apoptin and lipidosome/pUAS-Apoptin transfected cells exhibited a red color in the nucleus, indicating that these cells were dead and the membrane integrity was destroyed (Fig. 4h–j). Conversely, the G/pUAS-Apoptin and NG/pUAS-Apoptin transfected cells showed a green color in the core (Fig. 4a–g), indicating that these cells were alive. By staining with DAPI, the visualized nucleus was broken and disintegrated in TG/pUAS-Apoptin, TNG/pUAS-Apoptin and liposome/pUAS-Apoptin transfected cells, while the cell nuclei were integrated in the other groups.

**Fig. 5:**
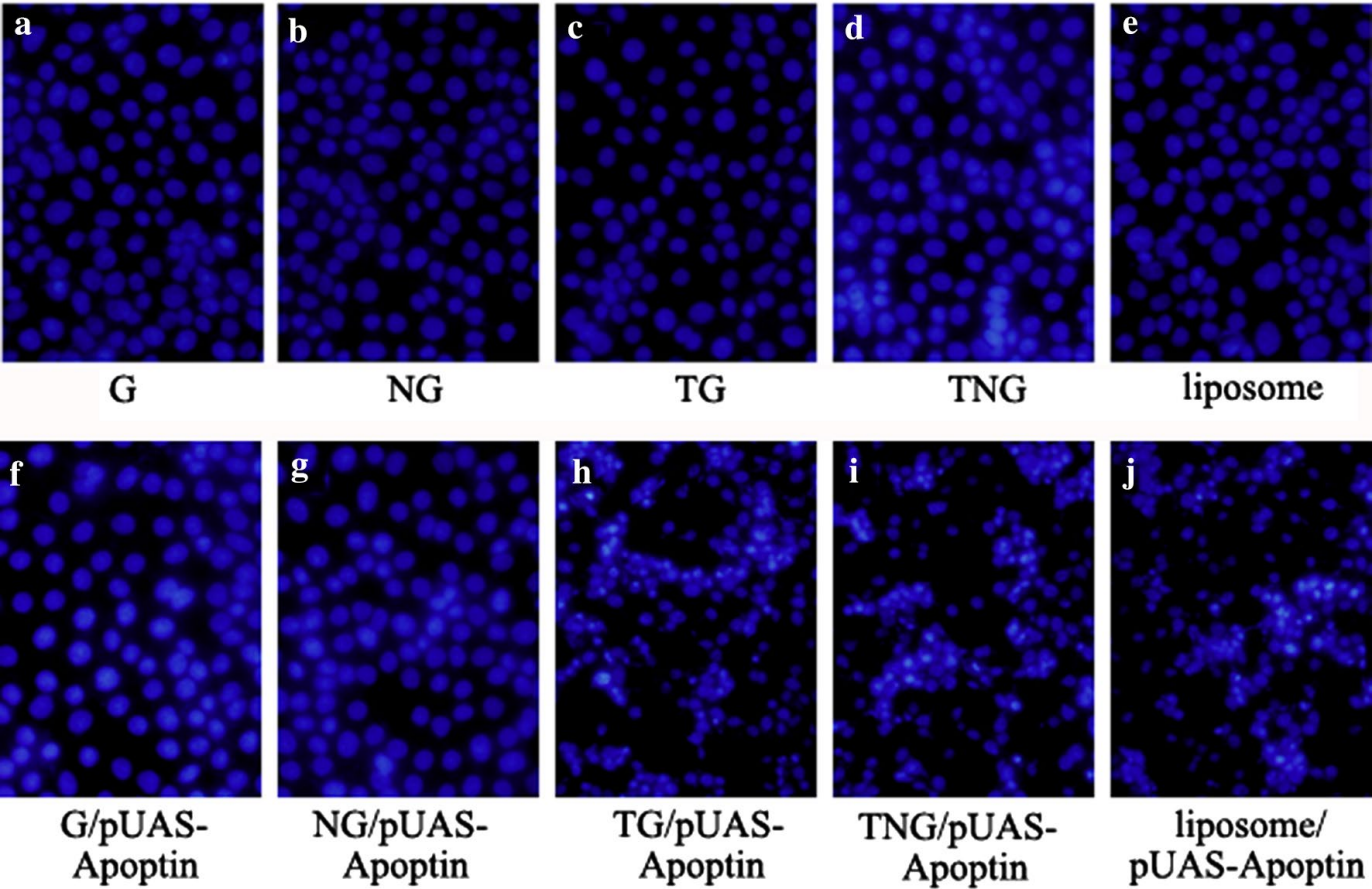
DAPI staining result of different treated HepG-2 cells (×100). The visualized nucleus was broken and disintegrated in TG/pUAS-Apoptin, TNG/pUAS-Apoptin and liposome/pUAS-Apoptin transfected groups, while they were integrated in the other groups. **a** G; **b** NG; **c** TG; **d** TNG; **e** liposome; **f** G/pUAS-Apoptin; **g** NG/pUAS-Apoptin; **h** TG/pUAS-Apoptin; **i** TNG/pUAS-Apoptin; **j** liposome/pUAS-Apoptin

The rates of cell death in TG/pUAS-Apoptin, TNG/pUAS-Apoptin and liposome/pUAS-Apoptin groups were 77.67, 72.67 and 64 %, respectively based on AO/EB staining (Table 1), while the apoptotic rates were 45.00, 36.00 and 61.33 % by DAPI staining (Fig. 5; Table 1). The cell apoptotic rates were significantly higher in TG/pUAS-Apoptin, TNG/pUAS-Apoptin and liposome/pUAS-Apoptin group than those in G/pUAS-Apoptin and NG/pUAS-Apoptin group (*P* < 0.05). These results indicated that pUAS-Apoptin could induce cellular morphology changes induced by cell death, when mediated by TG and TNG.

**Table 1:** The counts of apoptosis cells stained by AO/EB and DAPI staining

Groups	Apoptosis/necrosis cell number N = 300		Dead cell percentage (%) N = 300	
	AO/EB	DAPI	AO/EB	DAPI
G	17	26	5.67	8.67
NG	43	27	14.33	9.00
TG	28	14	9.33	4.67
TNG	37	31	12.33	10.33
Liposome	21	9	7.00	3.00
G/pUAS-Apoptin	36	12	12.00	4.00
NG/pUAS-Apoptin	51	25	17.00	8.33
TG/pUAS-Apoptin	233^a^	135^a^	77.67^a^	45.00
TNG/pUAS-Apoptin	218^a^	108^a^	72.67^a^	36.00
Liposome/pUAS-Apoptin	192^a^	184^a^	64.00^a^	61.33

^a^Indicates significant difference compared with G, NG, TG, TNG, liposome, G/pUAS-Apoptin and NG/pUAS-Apoptin, *P* < 0.05. Total cell number was 300

### TG and TNG mediated pUAS-Apoptin induced caspase activity

**Fig. 6:**
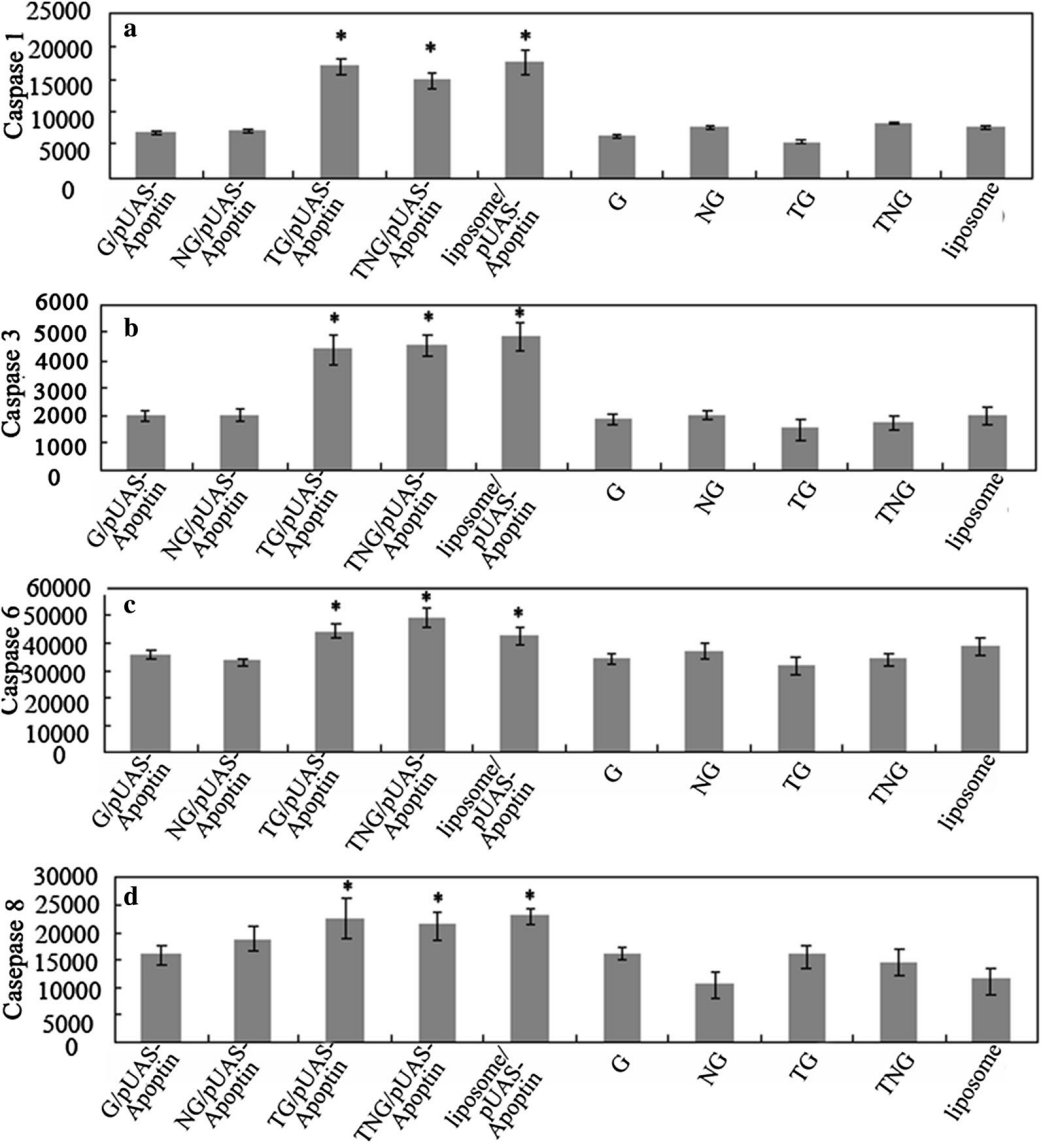
Caspase assay in different treated HepG-2 cells. The activity of Caspase 1, 3, 6 and 8 was significantly induced in TG/pUAS-Apoptin, TNG/pUAS-Apoptin and lipidosome/pUAS-Apoptin group. **a** Caspase 1; **b** Caspase 3; **c** Caspase 6; **d** Caspase 8. *Asterisk* indicates significant difference compared with G/pUAS-Apoptin, NG/pUAS-Apoptin, G, NG, TG and TNG treated cells at *P* = 0.05 level

To detect whether the caspase involved in the cell apoptosis of HepG-2 cells in different groups, the activity of caspase was detected. Figure 6 showed that the activity of Caspase 1, 3, 6 and 8 was significantly increased in TG/pUAS-Apoptin, TNG/pUAS-Apoptin and lipidosome/pUAS-Apoptin group.

### TG and TNG mediated pUAS-Apoptin inhibited tumor growth in H22 induced HCC mice

**Fig. 7:**
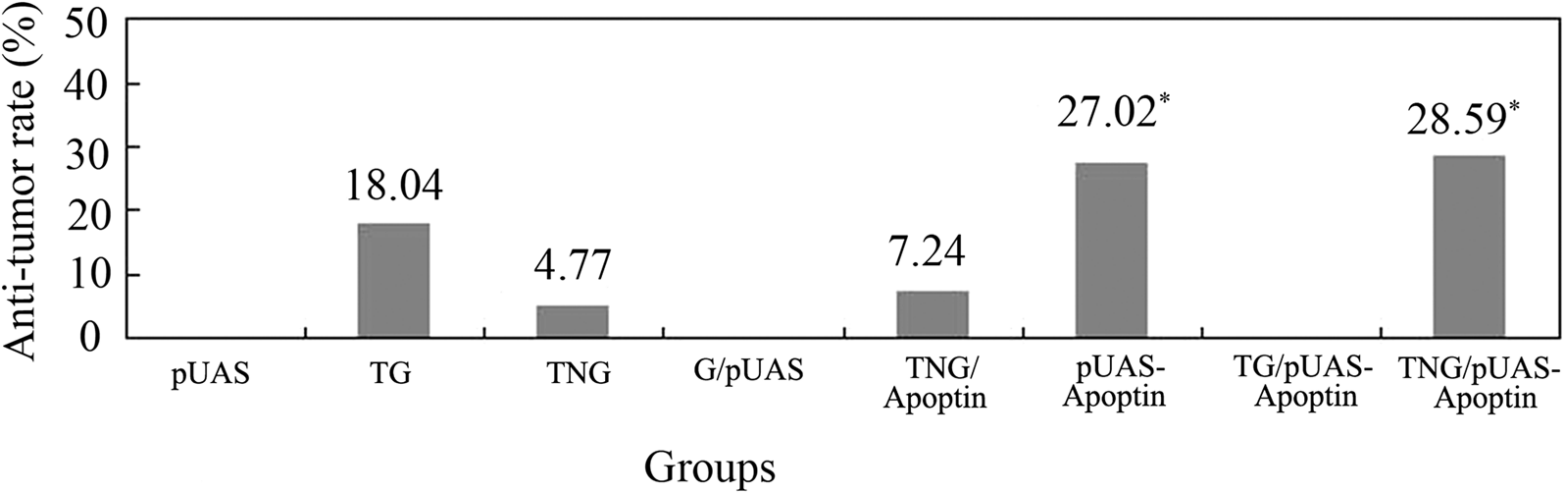
The tumor suppression rates in H22 induced HCC mice after pUAS-Apoptin plasmid transfection with different gene delivery vehicles

Tumor volume of mice in different treatment groups were recorded during treatment. As a result, the tumor volume was smaller in TG/pUAS-Apoptin and TNG/pUAS-Apoptin treated mice group than in the other groups at the same time point (Additional file 4: Table S1). Besides, the tumor suppression rates in TG/pUAS-Apoptin and TNG/pUAS-Apoptin groups were 27.02 and 28.59 %, respectively, which were higher than other groups (Fig. 7).

### pUAS-Apoptin elevated survival rate of HCC model mice when mediated by TG and TNG

**Fig. 8:**
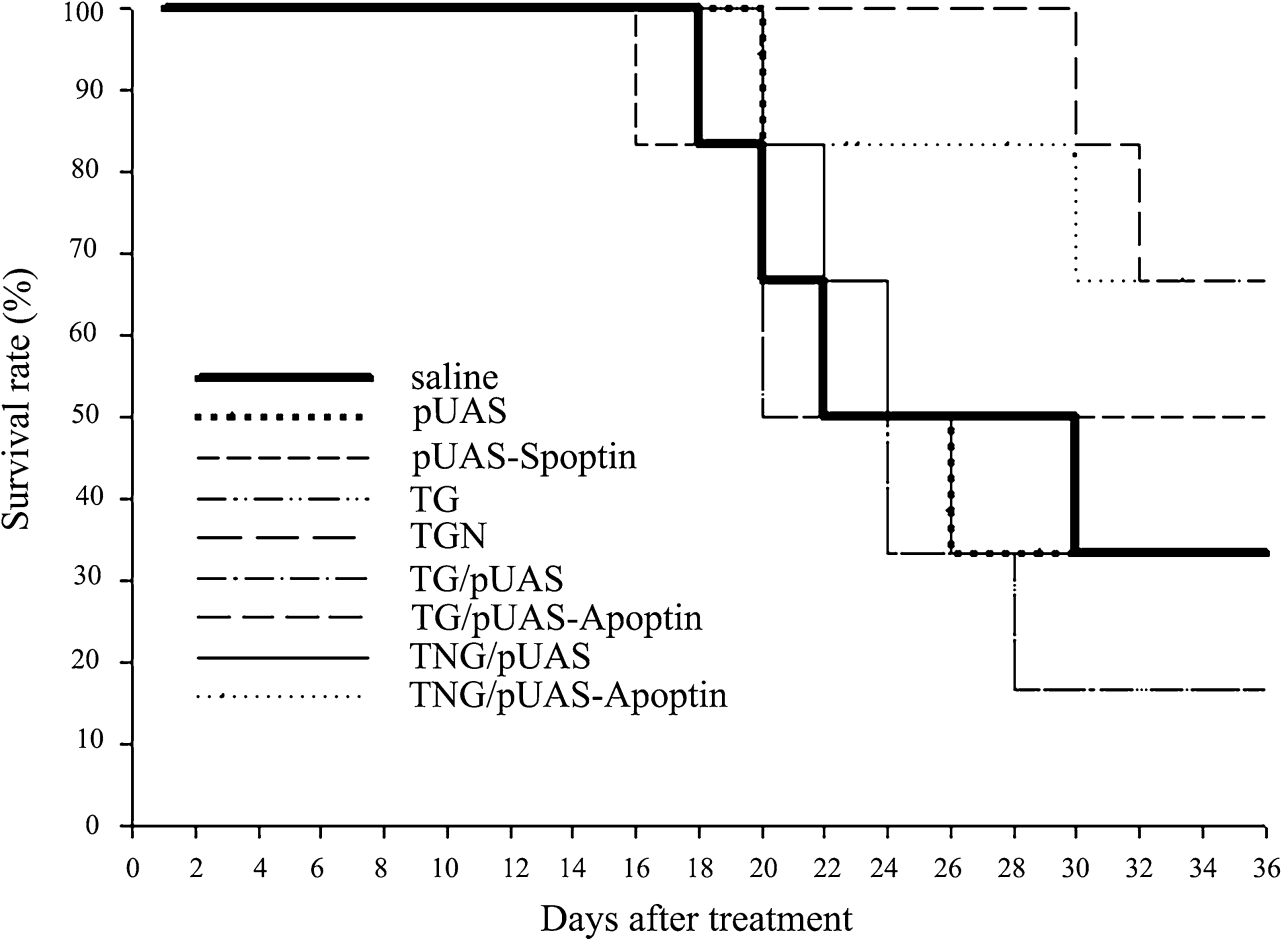
Kaplan–Meier curve for analysis of survival rate of different treated HepG-2 cells

Survival curve showed that the mean survival rates of HCC model mice treated by TG/pUAS-Apoptin (66.7 %) and TNG/pUAS-Apoptin (66.7 %) were relatively higher than those in the other treatment groups such as normal saline (33.3 %), pUAS (33.3 %), pUAS-Apoptin (50 %), TG (16.7 %), TNG (33.3 %), TG/pUAS (16.7 %) and TNG/pUAS (33.3 %) (Fig. 8).

### pUAS-Apoptin induced cell death in HCC model mice when mediated by TG and TNG

**Fig. 9:**
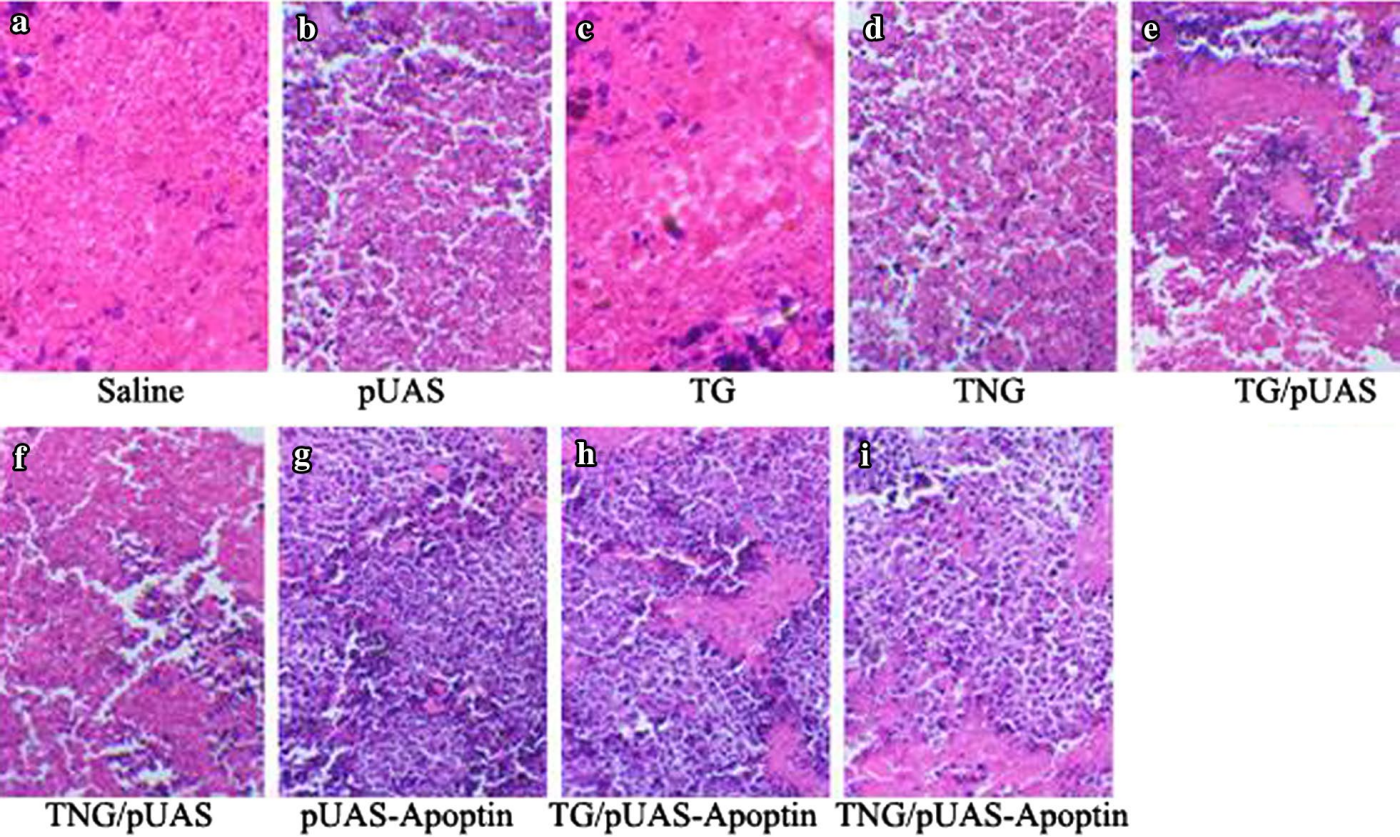
The pathological section assay stained by HE (×40). **a** saline; **b** pUAS; **c** TG; **d** TNG; **e** TG/pUAS; **f** TNG/pUAS; **g** pUAS-Apoptin; **h** TG/pUAS-Apoptin; **i** TNG/pUAS-Apoptin

HE staining results showed that the tumor cells obtained from mice treated by saline, pUA, TG, TNG, TG/pUAS and TNG/pUAS were normal and vigorous. The tumor tissue treated with TG/pUAS-Apoptin and TNG/pUAS-Apoptin and pUAS-Apoptin showed increased intercellular space, cell shrinkage and thick dyeing (Fig. 9).

## Discussion

In the present study, we designed several novel non-viral gene delivery vehicles (G, NG, TG, TNG) for pUAS-Apoptin plasmid transfection, and evaluated their combined therapeutic effect on human HCC in vitro and in vivo. As a result, the TG/pUAS-Apoptin and TNG/pUAS-Apoptin showed high therapeutic effect on promoting cell apoptosis by mediating the activity of Caspase 1, 3, 6 and 8 and reducing mortality of model mice.

After HepG-2 cells were transfected with pUAS-EGFP mediated by G, NG, TG and TNG, green fluorescence was only observed in HepG-2 cells transfected with TG/pUAS-EGFP and TNG/pUAS-EGFP for 48 h, as well as with the liposome/pUAS-EGFP, which suggested the pUAS-EGFP plasmid were transfered to cells effectively by TG and TNG delivery vehicles. In addition, the suppression rates of HCC cells were significantly higher in HepG-2 cells transfected with TG/pUAS-Apoptin, TNG/pUAS-Apoptin and lipidosome/pUAS-Apoptin than others and the cytotoxic effect of pUAS-Apoptin induced by TG and TNG was in a time dependent manner. All these suggested that the TG and TNG were the effective delivery system for bare plasmids, and the apoptosis rate of HepG-2 cells transfected with pUAS-Apoptin by TG and TNG was comparable to liposome delivery vehicle. As a small apoptosis-inducing protein, apoptin has attracted great interest because it can specifically kill tumor cells and leave the normal cells unharmed [22, 23]. Apoptin is found to be located in the cytoplasm of normal cells while mainly in nucleus of cancer cells [24]. This location is suggested to be the major mechanism of anti-tumor cell killing mediated by apoptin. Previous studies have demonstrated the selective anti-tumor activity of apoptin in several human tumor cells, such as melanoma, lymphoma, hematoma, colon carcinoma, and breast [25, 26]. In tumor cell selectivity, apoptin is able to trigger the Caspases independently, and this process is also proved to be independent of death receptors [27, 28]. High levels of apoptin in transformed cells activate the release of cytochrome *c* and Caspase 3 and -7 and result in a serious cell apoptosis which is not only involved with Bcl-2, caspase dependent pathways but also involved with the APAF-1 apoptosome-mediated mitochondrial death pathway [29, 30]. In addition, the apoptin induced cell death is intensively influenced by the regulators in mitochondrial pathway [29]. A study showed that when recombined into the JDK plasmid, apoptin gene could significantly reduce the tumor volume to 16 % of the control PBS-treated tumors in vivo [31]. The present study indicates that the activity of Caspase 1, 3, 6 and 8 was all significantly induced in TG/pUAS-Apoptin, TNG/pUAS-Apoptin and lipidosome/pUAS-Apoptin group. Besides, the tumor volume was smaller in TG/pUAS-Apoptin and TNG/pUAS-Apoptin treated mice group. When mediated by TG and TNG, pUAS-Apoptin significantly suppressed the tumor with anti-tumor rate of 27.02 and 28.59 % respectively.

Tat protein encoded by HIV-1 is a regulatory protein that promotes viral transcription and replication [32]. The capacity of Tat to enter cytosol has been used for protein imported to cells [33–35]. A minimum effective sequence, named Tat basic domain, is able to target attached proteins into the nucleus [17]. It was reported that when the basic domain was deleted or mutated, Tat was unable to be delivered to the nucleus [36, 37]. When attached to beta-galactosidase, the basic domain chimera is more efficiently to target to the nucleus [38]. Previous study has proved that the Tat-apoptin could migrate from the cytoplasm to the nucleus in Saos-2 and HSC-3 cells and then led to cell apoptosis [39]. In one of our previous studies, a combination of GAL4/147 with Tat (named as TG) has been constructed, which has been demonstrated as the efficient non-viral DNA transfer vectors for further improvements of gene therapy strategies [18]. These results were supported by the present results that TG and TNG protein successfully delivered pUAS-Apoptin plasmid into HCC cells in vitro and in vivo.

A novel gene transfer strategy is developed for the effective delivery system and the clinical practice [40, 41]. In spite of the effective mediation of Tat on gene transfer, the exogenous gene carried apoptin was also successfully expressed by regulating HCC cell death [42]. MTT assay in our study suggests that the TG and TNG mediated pUAS-Apoptin significantly induced cancer cell death in a time dependent manner. We then detected the cell death pathway by Annexin V assay, AO/EB and DAPI staining and Caspase activity, and found that the pUAS-Apoptin transfection mediated by TG and TNG in HepG-2 cells significantly induced cell apoptosis by increasing the activity of Caspase 1, 3, 6 and 8. The anti-tumor efficiency was also proved by the animal experiment on H22 induced liver cancer mice model. The mice in TG/pUAS-Apoptin and TNG/pUAS-Apoptin treated groups had a relatively higher anti-tumor rate and survival rate than that in the other groups, indicating TG and TNG were the effective delivery of pUAS-Apoptin plasmid in vivo. Based on a virus delivery of lentivirus (LV), Ma et al. [43] investigated the antitumor efficacy of secretory Tat-apoptin for HCC cells and found that most of the HCC xenograft tumors disappeared following the treatment of LV-secretory signal peptide (SP)-Tat-apoptin/GFP. Although the animal study has demonstrated a low toxicity of SP-Tat-apoptin in vivo, it may have potential toxicity in widely clinical application. Herein, with a design of a non-virus gene delivery based on Tat-apoptin system, our study may provide a safer and effective gene therapeutic strategy in vitro and in vivo.

Although the synthetic protein delivery carriers show advantages in flexibility in design and non-infectivity, whether TG and TNG affects the specificity of apoptosis induction in cancer cells vs. normal cells should be further validated. In addition, whether the non-viral delivery carrier improves the Apoptin expression should be further investigated.

In conclusion, we construct a chimeric multi-domain protein mediated specific DNA transfer, and apply it on HCC treatment in vitro and in vivo. The results strongly demonstrate that the systemic delivery of non-virus based Tat-apoptin is feasible in liver cancer treatment. Rigorous study is still needed for further verification of our results and fully understanding of corresponding mechanisms.

## Additional files

10.1186/s12935-016-0351-0 The structure of plasmid of pUAS.

10.1186/s12935-016-0351-0 The schematic representation of plasmid chimera and fusion protein transfection into human HepG-2 cells.

10.1186/s12935-016-0351-0 The suppression rates of HepG-2 cells at different time points.

10.1186/s12935-016-0351-0 The tumor volumes of HCC mice in different treatment groups.

## Abbreviations

HCC: human hepatocellular carcinoma
MTT: 3-(4,5-dimethylthiazol-2-yl)-2,5-diphenyltetrazolium bromide
AO: acridine orange
EB: ethidium bromide
DAPI: 4′,6-diamidino-2-phenylindole
HIV-1: human immunodeficiency virus type 1
NG: none GAL4
TG: GAL4 + Tat protein
TNG: Tat protein
PBS: phosphate buffer solution
FITC: fluorescein isothiocyanate
PI: propidium iodide
BCA: 2,2′-biquinoline-4,4-dicarboxylic acid disodium salt
ELISA: enzyme-linked immuno sorbent assay
HE: hematoxylin–eosin
