# Supplementary figures and images for: The chimeric multi-domain proteins mediating specific DNA transfer for hepatocellular carcinoma treatment
Source: Cancer Cell Int. 2016 Oct 13;16:80. doi: 10.1186/s12935-016-0351-0 (PMC5062862; doi:10.1186/s12935-016-0351-0)

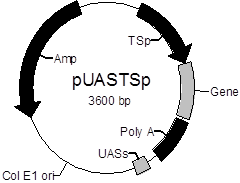

Supplement: Supplementary file 1 — 10.1186/s12935-016-0351-0 The structure of plasmid of pUAS. [file 12935_2016_351_MOESM1_ESM.tif]

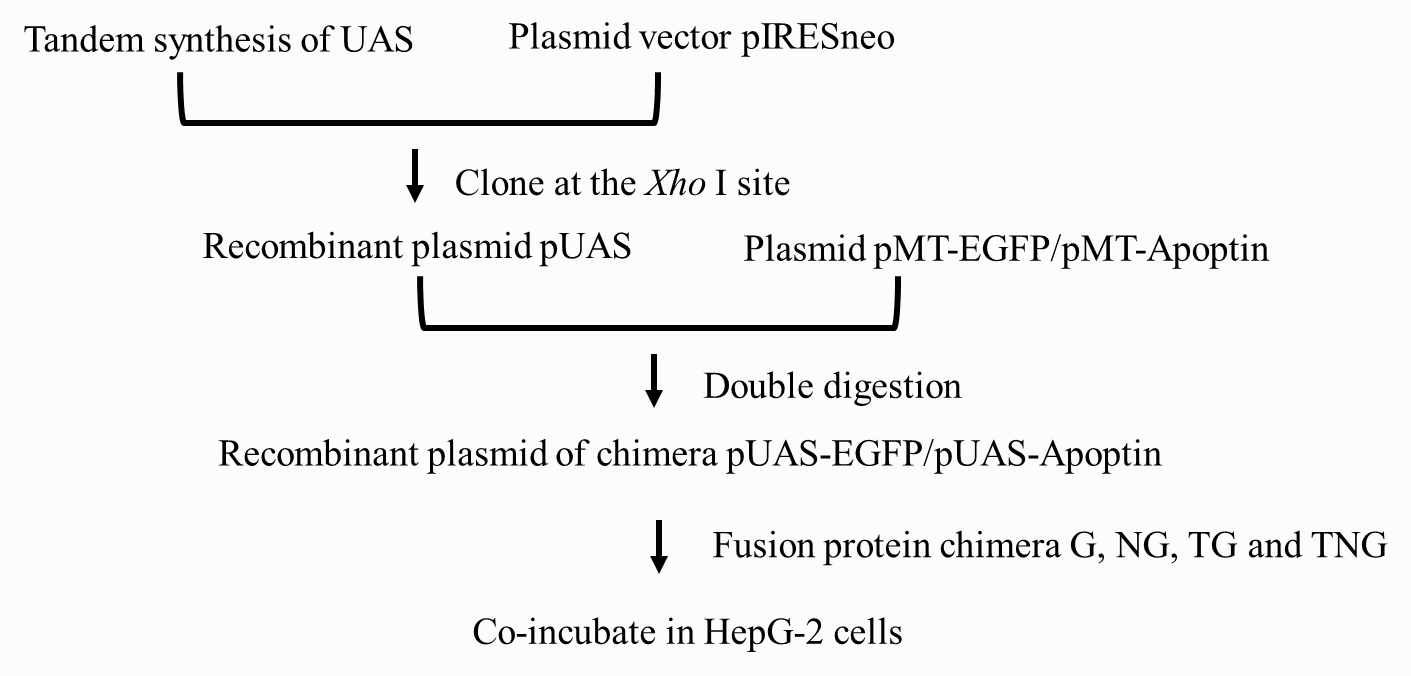

Supplement: Supplementary file 2 — 10.1186/s12935-016-0351-0 The schematic representation of plasmid chimera and fusion protein transfection into human HepG-2 cells. [file 12935_2016_351_MOESM2_ESM.tif]
